# Older African American Men’s Perspectives on Factors That Influence Type 2 Diabetes Self-Management and Peer-Led Interventions

**DOI:** 10.3390/geriatrics3030038

**Published:** 2018-07-06

**Authors:** Jaclynn Hawkins, Jamie Mitchell, Gretchen Piatt, Deborah Ellis

**Affiliations:** 1 School of Social Work, University of Michigan, Ann Arbor, MI 48109, USA; Mitchj@umich.edu; 2 School of Medicine, University of Michigan, Ann Arbor, MI 48109, USA; piattg@med.umich.edu; 3 School of Medicine, Wayne State University, Detroit, MI 48202, USA; dellis@med.wayne.edu

**Keywords:** diabetes, self-management, men’s health, African American

## Abstract

Older African American men are at increased risk of Type 2 Diabetes (T2D) but demonstrate high rates of poor illness management. They also participate in interventions targeting illness management at extremely low rates and are at high risk for dropout from clinical trials. One modifiable factor that has been identified in the literature that contributes to these disparities is health beliefs particular to men. Yet, despite the fact that illness management interventions have been developed to meet the needs of African Americans, none have followed recommendations to use gender-sensitive programming to meet the needs of men. The primary aim of this study was to advance our understanding of the intersection of age, race/ethnicity and gender on T2D self-management among older African American men, and to explore their preferences for a peer-led T2D self-management intervention. Two focus groups were conducted with older African American men (*n* = 12) over a 6-month period. Sessions lasted 90 min, were audiotaped, and analyzed using thematic content analysis techniques. The most prominent themes included: (a) the influence of gendered values and beliefs on health behavior; (b) quantity and quality of patient-provider communication; (c) social and structural barriers to T2D self-management; and (d) preferences for peer-led T2D self-management interventions. Results suggest that these themes may be particularly salient for T2D self-management in older African American men, and that this population may be receptive to a peer-led T2D self-management intervention.

## 1. Introduction

In the United States, about 30 million (or 12%) of adults over the age of 18 have diabetes. For adults over the age of 65, this percentage increases to 25% [1]. Differences in diabetes diagnosis also vary by gender and race—13% of black men have diabetes compared to black women, and black men have a 1.5 higher incidence of Type 2 Diabetes (T2D) compared to non-Hispanic White men. Black men in particular are twice as likely to die from diabetes-related complications and this disparity increases among men over the age of 55 [1]. Not following T2D management regimens dramatically increases the risk for heart disease and stroke, blindness and eye problems, kidney failure and lower limb amputation [1]. Black men have poorer glycemic control compared to non-Hispanic White men, and as a result, their risk for these diabetes complications is higher [2,3].

### 1.1. Barriers to T2D Illness Management among Older Black Men

One factor that may account for T2D health disparities are T2D self-management behaviors in Black men [4]. Although studies of older Black men are more limited, those that exist suggest that older Black men are even less likely to engage in diabetes self-management behaviors (e.g., checking blood glucose levels daily) [5]. Black men are also more likely than their White peers to engage in behaviors, such as smoking and alcohol consumption, which increase their risk for diabetes-related complications [6]. They may also face unique interpersonal and systemic barriers that interfere with access to or opportunities for engaging in healthy behaviors, including a lack of social support, negative patient-provider relationships, high medical fees, and long work hours [7,8].

A growing body of literature prioritizes the critical role of gender in the management of health behaviors that shape outcomes for chronic illnesses such as T2D. In the context of this discussion, sex refers to biological differences such as chromosomes, and sex organs while gender describes the characteristics that a society or culture delineates as masculine or feminine [9]. Studies show that traditional male gender norms can discourage healthy behaviors, with significant implications for the health trajectories of older Black men [10,11]. For instance, cultural expectations for male self-reliance can deter help-seeking from health professionals, and societal beliefs that men should display autonomy and dominance, and cope independently with pain or discomfort, can pose barriers to asking for help from family members to support health behavior change, or following health advice given by medical professionals [11]. Among Black men specifically, the need to exhibit toughness, confidence, to suppress emotions, and to appear independent and in control can serve as barriers to both accepting advice from care providers and accepting social support offered by family and community networks [12]. In a recent study, Black men reported encountering significant difficulty in accepting a caring environment, such as emotional support, as this was perceived as feminized behavior, which in turn served as a barrier to seeking care [3]. Gender-related norms among Black men also include “superman syndrome” or the ability to maintain their health without the assistance of doctors [13]. In another study, Black men with T2D reported waiting until symptoms became severe before seeking medical attention [10]. These findings are consistent with prior work, which examined the role of male gender norms in diabetes self-management for a sample of Black men diagnosed with T2D [5]. Results from that study aligned with the broader literature in showing that the need to maintain a strong image to the outside world by not acknowledging symptoms of illness, and to maintain control of one’s own health served as barriers to engaging in healthy behaviors—particularly seeking help from family members and attending doctor appointments [5]. These studies suggest that tailoring T2D self-management interventions to address the unique intersectional needs of age, gender, and race for men may be critical to helping them to achieve optimal health outcomes [5,10,11]. Such findings also suggest the need to tailor messaging for diabetes management for men to emphasize ways that caring for one’s own health and health care addresses values and goals particular to Black men (e.g., staying healthy allows one to continue to provide for one’s family).

### 1.2. Interventions to Improve Older Black Men’s T2D Management

While existing research clearly shows that Black men are at elevated risk for poor diabetes self-management, they are also less likely to participate in interventions to improve diabetes management [14]. Specifically, in a systematic review of T2D self-management interventions targeting Blacks, the percentage of male participants was typically less than 15%. This is also consistent with the finding that men are more likely to drop out of diabetes self-management intervention trials [11,14,15]. 

Community health workers are lay individuals who are trained to provide ongoing diabetes self-management support, particularly in minority communities. Community health workers are trained to assist individuals in goal setting and problem solving, and they provide social and emotional support for disease management [16,17]. Their shared cultural identity and community ties are a critical component in being perceived as trustworthy and accessible in their efforts to improve T2D management in minorities [17]. However, despite evidence that diabetes management interventions using community health workers have been effective, the majority of both community health workers and participants with T2D in those studies are women [17]. Research on Blacks with T2D found gender disparities in participation in diabetes self-management interventions, with men participating at significantly lower rates than women [11,18]. The limited studies to date suggest that Black men with T2D prefer peer-led T2D programs [17,19]. Use of lay interventionists to promote health behavior change in men is also consistent with approaches used with other populations managing chronic illness such as Black men with human immunodeficiency virus (HIV) [20].

Moreover, while studies showing that interventions using community health worker models have been successful in improving diabetes management (e.g., blood glucose monitoring) and clinical outcomes (e.g., HbA1c) among minority populations to date, these interventions have not been adapted to meet the needs of older Black men. In addition to the lack of tailoring of content or messaging, as noted earlier, the majority of community health workers used in these studies have been women [14]. There is a distinct gap in knowledge on the effectiveness of utilizing male community health workers with Black men with T2D; however, gender-matched lay-helper models have been used in other areas of health care to provide Black men with health education and support [21]. These interventions have included prevention of chronic illness (e.g., screening for hypertension) in intervention venues such as barbershops [22]. However, this research consists primarily of nonrandomized, small sample feasibility studies and therefore additional work needs to be done to establish the efficacy of these approaches [21,22]. In addition, as the behavioral targets of these programs were primarily health promotion and/or prevention behaviors such as increasing condom use and disease screening, and not management of a chronic disease, it is important to assess whether use of male peers as interventionists can improve health outcomes among men already living with a chronic illness.

In summary, older Black men are at increased risk of T2D but demonstrate high rates of poor illness management. They also participate in interventions targeting illness management at extremely low rates, and are at high risk for dropout from clinical trials. One modifiable factor that has been identified in the literature that contributes to these disparities is health beliefs particular to men. Yet, despite the fact that illness management interventions have been developed to meet the needs of Blacks, none have followed recommendations to use gender-sensitive programming to meet the needs of men. As previously noted, there is a dearth of efficacious diabetes management interventions for Black men. While the effectiveness of diabetes self-management intervention studies delivering diabetes-related education and support for short-term clinical, psychosocial, and behavioral improvements is well-established, Black men in particular are less likely to participate and are at a higher risk of drop-out from these studies [14]. As such, understanding gender beliefs and values among older Black men is critical to gaining a better understanding of how men of color with a chronic illness manage their health and interact with the health care system. 

## 2. Methods

### 2.1. Study Design

Our study utilized a phenomenological approach with a sample of Midwestern Black men over the age of 55 with T2D to explore how gender-related beliefs and values might influence health behavior; and to extract their perspectives on a specific lay helper diabetes self-management intervention.

We selected a phenomenological research method to capture the perspectives of older men who shared a common diagnosis of T2D. The goal of phenomenological research is to systematically explore participants’ perceptions of their personal and social world, often concerning specific events or experiences [23]. This exploration occurs with a population that shares a common characteristic(s), but are heterogeneous in other ways such as differences in marital status, employment and other sociodemographic factors. While focus groups were not stratified by other socio-demographic factors as stated, each focus group participant shared the same health condition and gender, aligning with the purpose of the present study: to gain a better understanding of how men experienced and perceived the impact of gender values and beliefs and other factors on their health behaviors and to gather their perspectives on a lay help diabetes self-management intervention.

### 2.2. Participant Characteristics 

A total of 12 Black men over the age of 55 participated in this study. A majority of the men who participated in the study reported that they were born in the same Midwestern state. Fourteen of the men were retired at the time of the focus group and a majority of the men were single. For a more complete description of participants, see Table 1. 

### 2.3. Participant Recruitment and Data Collection

Potential participants were mailed a flyer describing the research project and then received a follow-up phone call from a research assistant. The Michigan Center for the African American Aging Research Participant Resource Pool (MCUAAAR PRP) is a research volunteer registry and can be accessed by scholars conducting research on African Americans of 55 years of age and older who meet their study criteria [24]. Flyers describing the study were posted at a Detroit-based senior center and MCUAAAR PRP community-partners. Men with T2D from the MCUAAAR PRP were also called by study staff and invited to participate in the intervention. Interested men called a central office phone number where they were scheduled for screening. At the time of recruitment, there were a total of 1424 active PRP members and 60.1% of male PRP members had T2D [24].

This study utilized data from two focus groups to capture information from the 16 Black male participants. Focus groups were chosen because they can assist a group of individuals with meaningful similarities to share ideas in a low-pressure environment [25]. Focus group discussions can produce rich data because they are free-flowing and usually involve the participation of a somewhat homogenous group of persons who are not familiar with one another, potentially lowering inhibitions [25]. We sought to follow established methodological practices for conducting focus groups by including 6–10 people per group and ensuring that discussions were facilitated by a trained moderator who was tasked with eliciting different ideas and opinions from group members during the given time period using a semi-structured interview guide. For the present study, focus groups were conducted over a 3-month period in 2017; sessions lasted around 90 min and were audio-recorded. Groups were conducted in community settings (i.e., a community organization and a church) in Eastside and Southwest Detroit, Michigan and participants received a $60 cash incentive for their time and received snacks and beverages during the focus group. 

This study’s lead investigator moderated each focus group. The lead investigator is a person of color with significant experience working in Black communities, along with research protocol development and focus group facilitation techniques. Research assistants were also present at each focus group with the primary task of taking notes, and ensuring consent forms were signed and demographic data was collected. Written informed consent was obtained from each participant before each focus group and the moderator read through the informed consent with participants in Spanish or English. Demographic data were collected with an 11-item questionnaire at the beginning of each group (see Table 1 for selected demographics). 

The present analysis focuses on questions from the focus group interview guide sections titled “Men’s Health Care Related Behaviors and Beliefs and Men’s Preferences for Community Health Worker Interventions”. The questions posed in this section were:

- “When you were growing up, what did you learn about being a man from the men in your community?”
- “How has what you learned about being a man from men in your community impacted your decisions about your health?”
- “In your opinion, what kinds of things do you think a community health worker could do in your community to support men trying to manage their diabetes?”
- “What sorts of things would you rather a community health worker not do?”

The study team developed these focus group questions and they were informed by and designed to address specific gaps in the literature on this topic. 

### 2.4. Data Management and Analysis

Promptly following the focus group sessions, the research team debriefed preliminary themes and subthemes session that arose from the study participants and documented this discussion to be referenced at a later date. After each focus group was transcribed, a group-based approach was employed to organize the data and identify themes and subthemes [26,27]. This approach, referred to as the rigorous and accelerated data reduction (RADaR) technique, involves the creation of “…data tables that produce qualitative results quickly and rigorously for translation and dissemination to intended audiences” [27] (p. 78). In accordance with RADaR, first, our research team revisited the study’s research question and read the transcripts several times in a way that has been described as becoming familiar with the data. Second, the research team created a table with the following subheadings to organize the data: focus group guide question, text chunk, code, notes, and theme. Using this table, the research team organized each focus group transcript and selectively reduced the transcripts to only segments of text and reaching agreements on quotes that related directly to initial research questions. 

The data reduction process described above took place in three phases, and conformed to the inductive, phenomenological research methodology described in the literature [26,27]. Lastly, a codebook was created from the themes and codes that were agreed upon by the research team members. While our study was not intended to be generalizable to all populations of older Black men living with T2D, identified themes and subthemes can be used as a springboard for further examination of the impact of gender values and beliefs and other factors on to T2D self-management and perspectives on an intervention model. We discuss these findings below. 

## 3. Results

**Figure 1:**
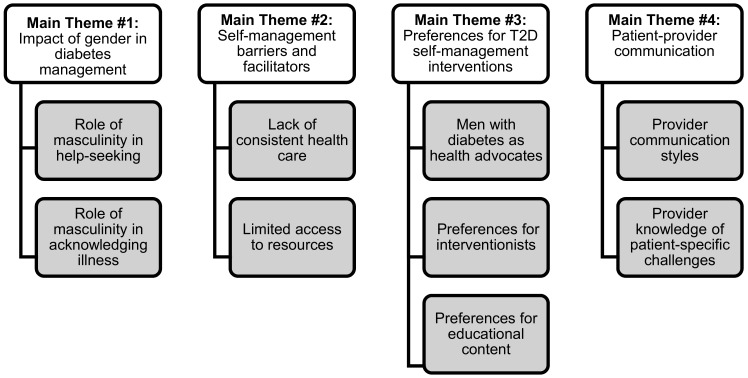
Main Theme and Sub-Theme Categories.

This study focused on explicating how a group of older Black men convened and engaged with one another around their gender-related beliefs and values in the context of influencing their diabetes-related health behaviors. Furthermore, this study elicited these men’s perspectives on T2D self-management using an evidence-based lay-helper diabetes self-management intervention model. Four themes arose from the data and are discussed in greater detail below: (1) impact of gender in diabetes management; (2) self-management barriers and facilitators; (3) men’s preferences for T2D self-management interventions; and lastly, (4) patient-provider communication. See Figure 1 for a thematic summary. 

Theme 1: Impact of Masculine Identity in Diabetes Self-Management.

Participants stated that their community norms of manhood reinforced that men should endure pain and avoid help-seeking, often resulting in delayed symptom reporting and avoidance of routine healthcare visits. Along the lines of this theme, one participant stated: “It’s a manly thing to put your foot in boiling hot water, and say it doesn’t hurt, and you know it does.” Yet another Black male participant discussed this theme by saying:

“When I found out I had cancer I got pretty scared and I ran for two years… without treatment. I went and had a biopsy … and he told me I had cancer. It was extremely aggressive and … I wasn’t playing superman, I was just scared. And I was scared to tell anybody that I was scared. Men ain’t scared, we’re superman and batman. I’ll beat up your Rottweiler.”

Constructions of masculinity for Black men in particular, have been studied extensively for the diagnosis and treatment decision-making surrounding prostate cancer [28] and HIV [29], as well broadly, in terms of both facilitators and barriers to health care access and utilization [30]. This theme in the current study underscores the need to examine the dynamics of culturally specific definitions of masculinity and the ways in which manifestations of masculine norms impact chronic disease diagnosis and self-management for medically underserved populations, such as Black men with Type 2 Diabetes.

Theme 2: Self-Management Barriers and Facilitators.

The second theme that emerged from coding this data concerned participants’ perceptions that they lacked consistent health care and had limited access to resources due to financial constraints. They reported that these limitations negatively impacted their ability to manage their health. For example, one participant stated:

“The assumption is that all I got to do is tell this gentlemen that ‘oh, you need a glucose meter,’ … but now what we forget [is] that people are living to eat whatever food they can really afford. Now, it may or may not be the appropriate food for a diabetic … we need to know what financial conditions … they have.” Yet another participant reflected on his experiences with accessing consistent care, stating, “and every time I went to the doctor, it was so frustrating. Every time I went to that clinic I had a different doctor.” Other in-depth qualitative studies have documented that consistency in interacting with the same health provider is both perceived as a hallmark of patient-centered care and is reported to be an important motivator for medically underserved patients to adhere to preventive and other treatment recommendations for managing their conditions [31].

Theme 3: Men’s Preferences for T2D Self-Management Interventions

In these focus groups with African American men living with Type 2 diabetes, we learned that men expressed a desire for men with diabetes to work as health advocates among their friends and family in an effort to share information about diabetes prevention and treatment. For example, one participant stated: “I urge all my children, my friends … make sure you keep your blood levels down … we become ambassadors. Six becomes twelve, twelve becomes and so on.” 

In expressing their preferences for individuals delivering T2D interventions and content, Black men in this study stated that they would like to learn about how to interact better with their physicians. These men reported that they would like to work with someone who understands not only diabetes but also their social situations. As an example, one participant stated: “That would be the important part to have somebody to further my knowledge of what I should focus on when I go in to see my doctor, what to ask, what to say, concerning my particular situations. That would really be interesting.” Yet another participant expressed, “They need to have an understanding of the person’s environment, and their social situation.” Here, participants were emphasizing the importance of shared experiences, possibly including shared circumstances, with the individuals who advised them on managing their T2D. As mentioned earlier, participants discussed feeling a disconnect between the recommendations provided by health providers on how to manage their condition, and the material resources they had at their disposal to adhere to the complex medical and behavioral regimen. 

Theme 4: Patient-Provider Communication

The final theme centered on black men’s experiences with patient-provider interactions, and how impactful positive communication and rapport, or a lack thereof, were on men’s diabetes self-management efforts. For instance, one participant shared the following about his patient-provider interactions: “just be concerned, without sugar coating. You can show a person that you care about them and that helps without being phony. And you ask relevant questions. And please do not scold me. Oh my god. Don’t do that ... Get off of my back. Leave me alone. So, you want a doctor who is kind, and caring, and concerned about you.” 

Men in this study also highlighted the need for an interdisciplinary team to be involved in managing their care: “you need a cardiologist, an internist, and a urologist. You need those, three amigos, I call them.” In line with these discussions, previous studies accentuate the need to focus on how African American men navigate difficulties in communicating with their healthcare team [32]. Men also discussed the need for health care professionals to have an in-depth understanding of the health risks specifically and uniquely burdening older Black men. One participant stated: “so the bottom line is that physicians if they know they are going to be working with older, African American men, they need to be educated about the cancer, the diabetes, and the other diseases that affect these men more than other groups of men.”

## 4. Discussion

Taken together, the themes that emerged from this qualitative analysis—centered on gender norms, T2D self-management barriers and facilitators, preferences for T2D intervention, and patient-provider communication—reinforce that Black men with T2D have specific unmet health needs and preferences for engaging with the management of their condition, and the health providers who support them. Given that diabetes self-management interventions to date have rarely targeted older Black men, who are at highest risk for poor diabetes outcomes, the themes emerging from this study underscore the potential need for peer-led T2D interventions with tailored intervention content to meet the specific needs of older Black men. Themes arising from these focus groups also underscore the need to equip Black men with the tools and support to engage in effective communication with medical providers in the context of their chronic disease management; communication has not been a target in T2D peer interventions to date. 

The ultimate aim of our research is that it be used to inform the development of tools and interventions designed to improve T2D self-management among Black men. Future research looking to extend this line of inquiry should center on developing and adapting trainings for gender-matched peer-leaders or lay helpers. Interventions should focus on how to encourage conversations between men and peer-leaders regarding beliefs that affect men’s health behaviors and consequent outcomes, along with how to best model alternative views and perspectives that allow for successful disease management to be framed as competence and strength, instead of weakness. 

Our study has limitations. First, while our findings may apply to populations of older Black men with T2D, of comparable communities and backgrounds, we cannot generalize these findings to all older Black men with T2D. However, our study may well be used in future research, with more complex analysis and larger numbers of older Black men, to further describe factors that impact T2D self-management for this population. Second, since our study focused on older men, our findings may not represent the experiences of younger Black men with T2D. While limitations do exist, this preliminary study is a critical first step in expanding the knowledge base on Black men’s T2D self-management. These limitations notwithstanding, our findings raise important issues with regard to Black men as a medically underserved population. The contribution of this research is to offer a more nuanced understanding of the unmet T2D self-management needs, preferences, and barriers facing this population, who have not traditionally been at the center of diabetes intervention efforts. 

## Figures and Tables

**Table 1:** Sociodemographic characteristics of Older African American men with type 2 diabetes (*n* = 12).

	African American Men(*n* = 12)
Education	
High School Diploma	1
Some College or More	11
Employment	
Employed	0
Not Employed/Retired	12
Marital Status	
Not Married	1
Married	8
Divorced/Widowed	3
Average Age	75
